# Stable isotope and chemical inhibition analyses suggested the existence of a non-mevalonate-like pathway in the yeast *Yarrowia lipolytica*

**DOI:** 10.1038/s41598-021-85170-0

**Published:** 2021-03-10

**Authors:** Sivamoke Dissook, Tomohisa Kuzuyama, Yuri Nishimoto, Shigeru Kitani, Sastia Putri, Eiichiro Fukusaki

**Affiliations:** 1 Department of Biotechnology, Graduate School of Engineering, Osaka University, 2-1 Yamadaoka, Suita, Osaka 565-0871 Japan; 2 Graduate School of Agricultural and Life Sciences, The University of Tokyo, 1-1-1 Yayoi, Bunkyo-ku, Tokyo, 113-8657 Japan; 3 International Center for Biotechnology, Osaka University, 2-1 Yamadaoka, Suita, Osaka 565-0871 Japan

**Keywords:** Microbiology, Metabolomics, Biochemistry, Metabolomics

## Abstract

Methyl erythritol phosphate (MEP) is the metabolite found in the MEP pathway for isoprenoid biosynthesis, which is known to be utilized by plants, algae, and bacteria. In this study, an unprecedented observation was found in the oleaginous yeast *Yarrowia lipolytica,* in which one of the chromatographic peaks was annotated as MEP when cultivated in the nitrogen limiting condition. This finding raised an interesting hypothesis of whether *Y. lipolytica* utilizes the MEP pathway for isoprenoid biosynthesis or not, because there is no report of yeast harboring the MEP pathway. Three independent approaches were used to investigate the existence of the MEP pathway in *Y. lipolytica*; the spiking of the authentic standard, the MEP pathway inhibitor, and the ^13^C labeling incorporation analysis. The study suggested that the mevalonate and MEP pathways co-exist in *Y. lipolytica* and the nitrogen limiting condition triggers the utilization of the MEP pathway in *Y. lipolytica*.

## Introduction

**Figure 1:**
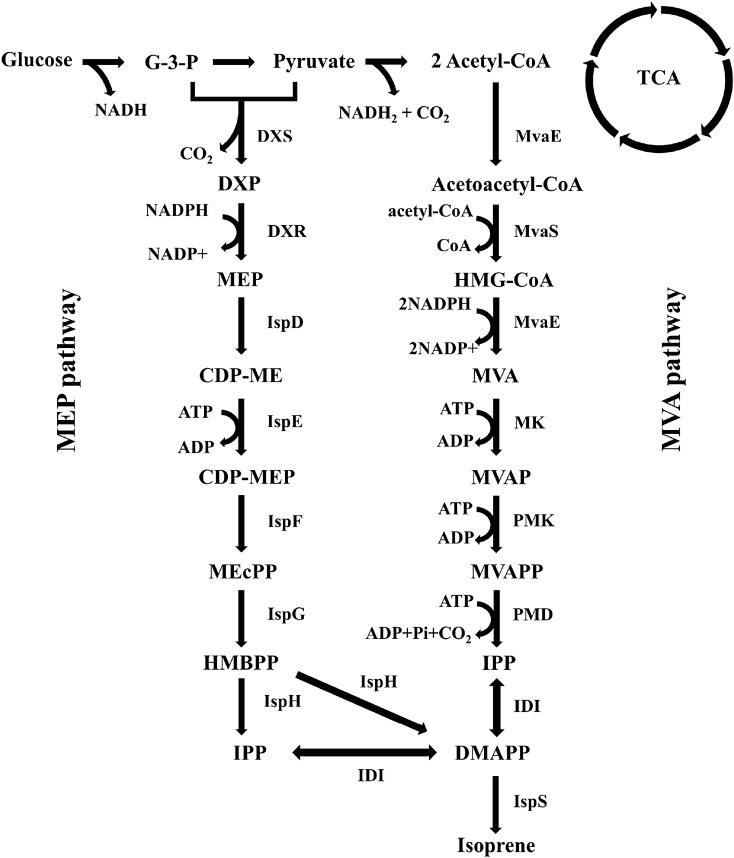
Biosynthetic pathways for isoprenoid precursors. The mevalonic acid (MVA) pathway in animals, plants (cytosol), fungi, and archaea. The methylerythritol phosphate (MEP) pathway in eubacteria, green algae, and the plastids of higher plants. Abbreviations: CDP-ME, methylerythritol cytidyl diphosphate; DXP, 1-deoxy-D-xylulose 5-phosphate; DXR, DXP reductoisomerase; DXS, DXP synthase; HMBPP, 4-hydroxy-3-methyl-butenyl-1-diphosphate; IPP, Isopentenyl diphosphate; IDI, IPP:DMAPP isomerase; MEcPP, 2-*C*-methyl-D-erythritol-2,4-cyclodiphosphate; MK, mevalonic acid kinase; PMD, phospho-mevalonate decarboxylase; PMK, phosphomevalonate kinase; DMAPP, dimethylallyl diphosphate; HMG-CoA, hydroxymethylglutaryl-CoA; MVA, mevalonic acid; MVAP, phosphomevalonic acid; MVAPP, diphosphomevalonic acid.

Isoprenoid compounds are the most diverse group of natural products with a broad range of biological functions^1^. All isoprenoids derived from isopentenyl diphosphate (IPP) and dimethylallyl diphosphate (DMAPP)^2^. In nature, two routes are leading to IPP and DMAPP; the mevalonic acid (MVA) pathway and the methylerythritol phosphate (MEP) pathway^3–5^ (Fig. 1). The MVA pathway is distributed among archaebacteria, a few eubacteria, and in the cytosol of almost all eukaryotic cells. In contrast, the MEP pathway is discovered in many bacteria, algae, cyanobacteria, apicomplexan parasites, and plant chloroplasts^6^. Although many studies reported that the MEP pathway does not exist in the yeast, surprisingly, a chromatographic peak annotated as MEP was observed in the intracellular extract of *Y. lipolytica* cultured in a nitrogen limiting condition. This phenomenon is inconsistent with a conventional understanding regarding isoprenoid biosynthesis in yeast.

The oleaginous yeast *Y. lipolytica* is an industrial model organism for the production of bio-sustainable hydrocarbon-based chemicals^7–13^. *Yarrowia lipolytica* is one of the most contrasting of the characterized Hemiascomycetes^14^. In spite of a genome nearly twice the size of *Saccharomyces cerevisiae*, *Y. lipolytica* is not thought to have undertaken whole-genome duplication^15^. Besides, *Y. lipolytica* has more characters shared with metazoan cells than other yeasts. These include signal-recognition-particle type 7SL RNA sequence, dispersed 5S genes, and a higher fraction of the genome composed of introns and intergenic sequences^14,15^. The *Y. lipolytica* genome also holds representatives of diverse classes of transposable elements, including remnants of a DNA transposon^16^, long-terminal repeat (LTR)^17^, and non-LTR Long INterspersed Element (LINE)^18^ retrotransposons. Lastly, contrasting to the more well documented respiro-fermentative *S. cerevisiae*, *Y. lipolytica* stands an obligate aerobe. *Yarrowia lipolytica* metabolizes a varied type of carbon substrates, including lipids, paraffin, oils, acetate, and glycerol, and is capable of accumulating a high ratio of cell weight in lipid^7,13,19–21^.

This study raises an intriguing hypothesis of whether *Y. lipolytica* could utilize the MEP pathway for isoprenoid biosynthesis. Here, we present a more in-depth investigation of the existence of the MEP pathway in *Y. lipolytica* using three independent approaches; the spiking of the authentic standard, the inhibition of the pathway-specific inhibitor, and analysis of the incorporation of the stable carbon isotope in the natural product. This study suggested that the MVA and MEP pathways co-exist in *Y. lipolytica*.

## Results

### MEP detection and verification

**Figure 2:**
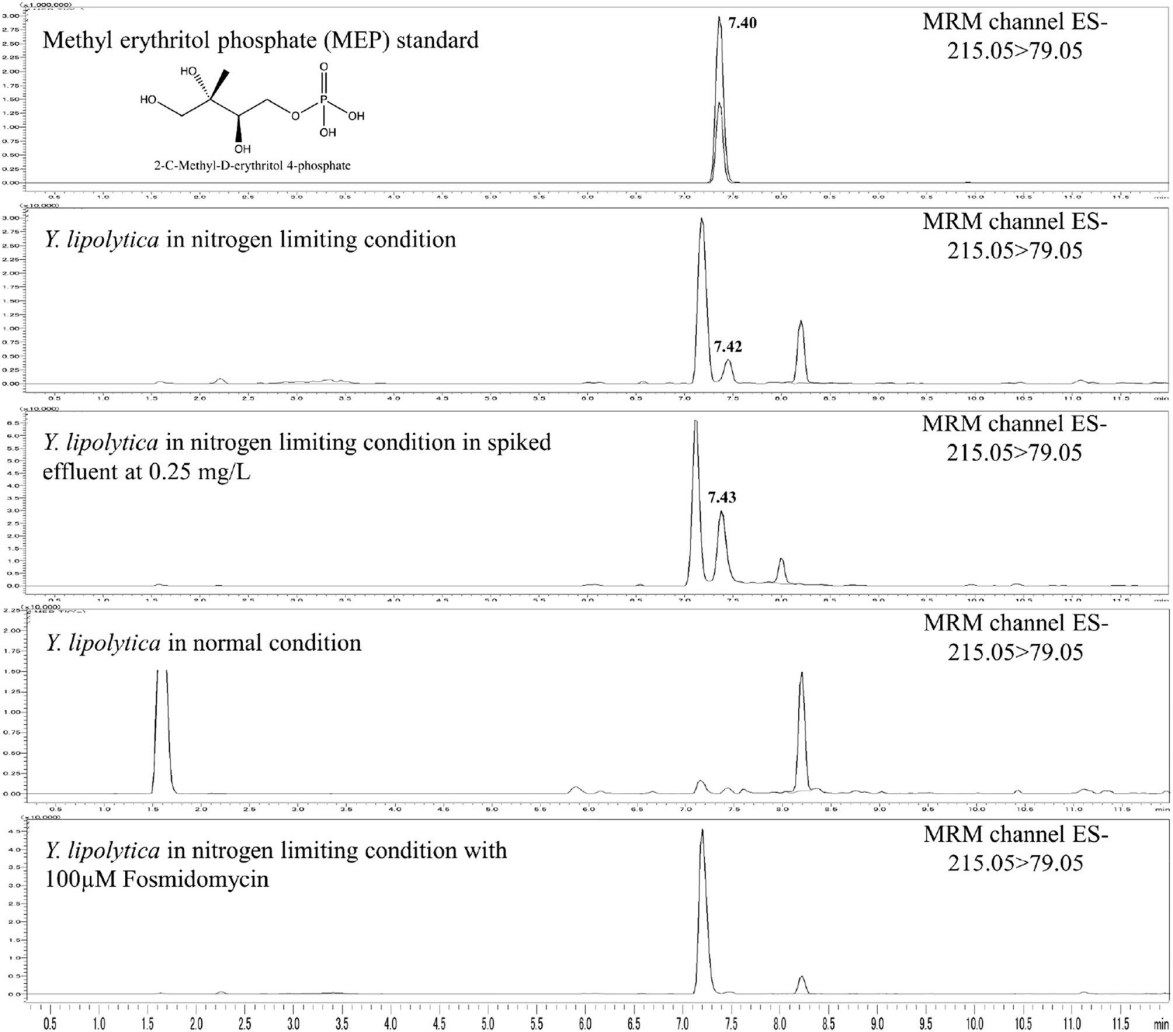
LC/QqQ/MS analysis results; Multiple reaction monitoring (MRM) chromatograms of methyl erythritol phosphate (MEP) standard, *Y. lipolytica* in nitrogen limiting condition, *Y. lipolytica* in nitrogen limiting condition in spiked effluent at 0.25 g/L, *Y. lipolytica* in normal condition and *Y. lipolytica* in nitrogen limiting condition cultivated with 100* µM* fosmidomycin.

The annotated MEP peak from the liquid chromatography with tandem mass spectrometry (LC/QqQ/MS) was found only in the sample from the culture extract of *Y. lipolytica* in which the nitrogen source was limited. Interestingly, the chromatographic feature was similar to the previous report on the MEP analysis in *E. coli,* where a larger peak with the same MRM was detected next to the MEP peak^22^. To confirm that the possible MEP chromatographic peak found in *Y. lipolytica* is comparable to the analytical standard; the spiking analysis of the authentic MEP standard was carried out. The results showed that the annotated MEP peak detected in *Y. lipolytica* was identical to the analytical MEP standard, thereby meeting the criteria for metabolite identification based on The Metabolomics Standards Initiative (MSI) for level 1 identification (Fig. 2). We also analyzed the sample in different LC/QqQ/MS systems (Supplementary Fig. 1).

### Effect of MEP pathway inhibitor

The antibiotic fosmidomycin, originally isolated from *Streptomyces lavendulae*, has been shown to inhibit bacterial isoprenoid biosynthesis^23^. It was later demonstrated that fosmidomycin specifically inhibits 1-deoxyxylulose 5-phosphate (DXP) reductoisomerase (DXR)^24,25^, which catalyzes the conversion of the DXP to MEP in the specific step of the MEP pathway. In this study, *Y. lipolytica* cultivated in nitrogen limiting condition was treated with fosmidomycin and analyzed with LC/QqQ/MS. The data show that the MEP peak that was elucidated with the authentic standard spiking earlier was reduced in the presence of fosmidomycin. The result suggested that DXR-like activity was present in *Y. lipolytica* and inhibited by fosmidomycin to have reduced the MEP content (Fig. 2).

### 1-^13^C carbon isotope glucose labeled experiment

**Figure 3:**
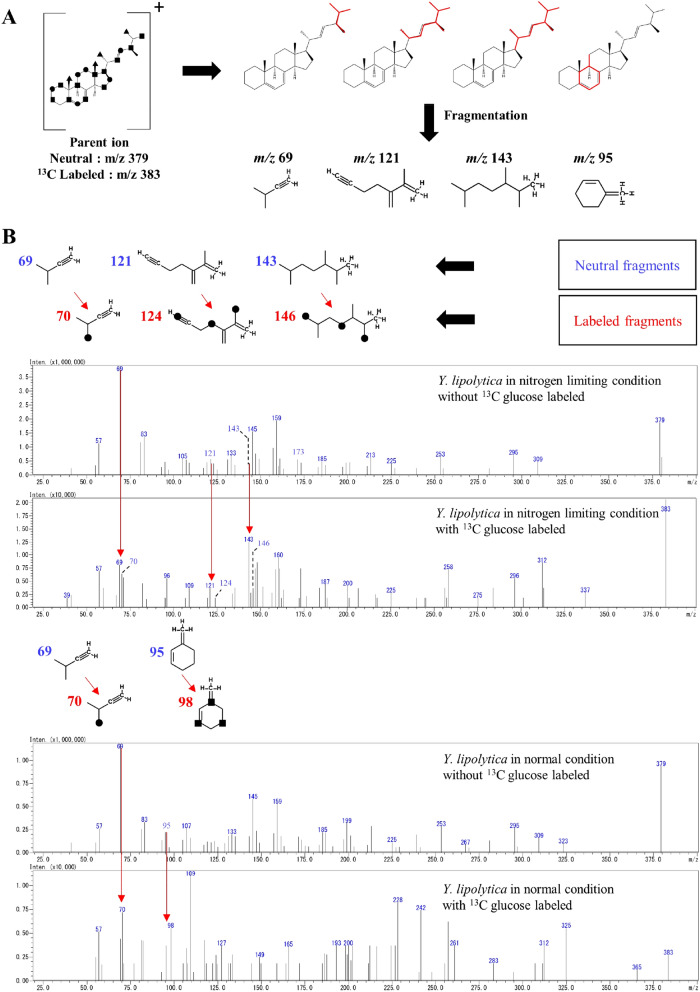
Mass spectroscopic analysis of ^13^C labeled ergosterol. (**A**) the predicted fragment from parent ion; (**B**) the mass spectra comparing the neutral and the labeled ergosterol product ion. The assign fragment structure elucidates the *m/z* increased specifically to the MVA pathway or the MEP pathway; circles indicate the theoretically labeled position from the MEP pathway, squares indicate the theoretically labeled position from the MVA pathway. All fragment structure assignment was done using CFMID 3.0 software. The predicted *m/z* and the structure of fragments resulted from the MEP pathway and the MVA pathway are found in Supplementary Fig. 4.

To further assess whether the MEP-like pathway works in *Y. lipolytica*, we examined the natural end product derived from DMAPP, which is the known product from both the MVA pathway and the MEP pathway. Ergosterol, which acts as a membrane stabilizer in yeast, is one of the most abundant natural products derived from IPP and DMAPP^26,27^. It has been established that incorporation of the 1-^13^C-labeled glucose through the MVA pathway and the MEP pathway resulted in the different labeling patterns^4^ (Supplementary Fig. 2). With this information, we are able to determine the theoretically labeled carbon position of the ergosterol from the MVA pathway and MEP pathway (Supplementary Fig. 3). In this study, LC/QqQ/MS was utilized to investigate the biological pathway involved in the biosynthesis of the ergosterol from *Y. lipolytica* cultivated in some conditions. All mass spectral data were extracted at 7.7 min, where ergosterol is eluted. Usually, the largest fragment of ergosterol detected in LC/QqQ/MS has a mass to charge ratio (*m/z*) of 379^28^. When 1-^13^C-labeled glucose was added to the culture medium of *Y. lipolytica*, the incorporation of the ^13^C carbon isotope increased the *m/z* of the fragment to the maximum of 383. Thus, this fragment of *m/z* 383 was selected as the parent ion for the product ion scan (Fig. 3A). Using CFMID 3.0, a mass spectra prediction software^29^, we were able to computationally assign the chemical structure to the ergosterol product ion generated by fragmentation of the parent ion. The full product ions are listed in Supplementary Table 1. By comparing the neutral fragment *m/z* and the labeled fragment *m/z*, it was possible to predict if the ergosterol were synthesized from the MVA pathway or the MEP pathway (Fig. 3B). In the condition where nitrogen source was limited, three fragments mass shift were found to match those of the peaks presumably derived from the MEP pathway; *m/z* 69 to 70, *m/z* 121 to 124, and *m/z* 143 to 146, whereas, in the normal condition, one fragment was found to be theoretically derived from each pathway; *m/z* 69 to 70 from the MEP pathway and *m/z* 95 to 98 from the MVA pathway. The predicted *m/z* and structure of fragments resulted from the MEP pathway, and the MVA pathway could be found in Supplementary Fig. 4. The fragment data show that ergosterol from the two conditions has a different *m/z* shift; this might be the result of the biosynthesis in different pathways. Combined with other results mentioned above, we concluded that ergosterol in *Y. lipolytica* might be derived from both the MVA pathway and the MEP pathway.

## Discussion

It is a common knowledge that altering the carbon to nitrogen ratio in culture medium could drastically change the amount of lipid accumulated in the oleaginous yeasts, and it might have a different effect on different strains of yeast ^30–33^. Substantial work has been done in the past few years to understand and engineer oleaginous yeast^34^. However, only limited work has been done to characterize the metabolic response of *Y. lipolytica* in nitrogen limiting condition. One of the best methods to investigate the cell’s response is by metabolomics approach. Metabolites are end products of a complicated network of reactions in which many regulatory processes (genes, mRNAs, enzymes, metabolites, etc.) are included; these data are known to possess a high correlation to a physical phenomenon^35^. Upon our data analysis, we stumbled upon a very startling data where one of the chromatographic peaks was annotated as MEP. Although the LC/QqQ/MS annotated data are widely accepted in the field of metabolomics due to its selectivity and sensitivity^36^, we took extra steps to verify the results by further checked the annotation with the authentic standard spiking technique. The spiking of the authentic standard provided the analytical chemistry evidence of the existence of the MEP pathway enzyme(s) in *Y. lipolytica.* Note that in the nitrogen limiting condition, another peak around 7.40 min that also only occurs in the nitrogen limiting condition, this might be a compound that is important for adapting to the nitrogen limiting condition or related to this MEP-like pathway. It would be interesting for future studies. The next issue we address is the evidence from the biological perspective. One of the best ways to assess the MEP pathway activity is to evaluate the enzyme specific to the MEP pathway; the DXP reductoisomerase^37^. However, no DXR gene has been annotated in the genome of *Y. lipolytica* and the BLAST homology search^41,42^ returns no significant alignment. We further investigated this issue with an in-house bioinformatics pipeline (Supplementary Fig. 5) and got some gene candidates. Nevertheless, a vital element of the known DXR gene was still lacking (Supplementary Fig. 1). The lack of the DXR gene information might be due to the lack of data on the laboratory-tested gene function as more than half of *Y. lipolytica* gene was not annotated^38^. We also hypothesized that *Y. lipolytica* might utilize an isozyme that catalyzes the DXR-like activity^39,40^ or that the MEP found in *Y. lipolytica* was synthesized by an enzyme with extremely low or no homology with the known enzyme in the MEP pathway. Additionally, to provide more information regarding the potential MEP pathway gene target in *Y. lipolytica*, the RNA sequencing data from a previous publication^44^ was analyzed (Supplementary Fig. 6) to create a list of candidate MEP pathway enzymes in *Y. lipolytica*. The detailed workflow^45–49^ could be found in Supplementary Fig. 2. The findings that *Y. lipolytica* is able to biosynthesize MEP, and the strain has the DXR-like activity could lead to a hypothesis that a pathway similar to the MEP pathway is present in *Y. lipolytica*. To assess this hypothesis, we performed an incorporation analysis with 1-^13^C-labeled glucose into ergosterol; which acts as a membrane stabilizer in yeast and is a natural product derived from IPP and DMAPP. There are several reasons why we chose ergosterol; 1. ergosterol was found accumulating in the yeast cell membrane with relatively high abundance (1–3% dried cell weight), 2. the ergosterol molecule provide multiple fragments that could be analyzed for the pathway differences, and they provide more mass different; therefore, the effect of the MEP or MVA pathway can be clearly observed, 3. the extraction and purification method for ergosterol was well established. This analysis was grounded on the fact that these building blocks are biosynthesized by completely different routes, the MVA pathway, and the MEP pathway (Supplementary Fig. 2)^4^. Although, this experiment was done with the assumption that the known MEP pathway was utilized in *Y. lipolytica*, the fragments found from the experiment match perfectly with the predicted mass shift. Moreover, the fact that the fragment mass shift from different conditions shifted differently could be an indication that different biosynthesis pathways were utilized. Combined with other results mentioned above, we concluded that ergosterol in *Y. lipolytica* might be derived from both the MVA pathway and the MEP-like pathway. Although more direct evidence such as the identification of all intermediates and discovery of key enzymes are still needed, the observations in this study provide important information for researchers to further verify the existence of the MEP-like pathway.

Altogether, our results suggested that the MVA pathway and the MEP-like pathway co-exist in *Y. lipolytica*. Notably, the nitrogen limiting condition triggered the utilization of the MEP-like pathway. Three types of evidence support the conclusion that *Y. lipolytica* might possess an enzyme(s) involved in the MEP-like pathway. Firstly, the authentic standard confirmed that the MEP was detected in *Y. lipolytica*. Secondly, the MEP peak was affected by the specific MEP pathway inhibitor fosmidomycin. Finally, the ^13^C labeled glucose feeding showed that the ergosterol fragments were matched with the theoretical calculations of the mass increased from both the MVA pathway and the MEP pathway. The genes involved in the MEP synthesis in *Y. lipolytica* and the mechanism on how nitrogen limiting conditions trigger this phenomenon remain unclear. Our findings reported here may spark new investigations on the possibility of the MEP pathway-like existence in yeasts that may be activated in specific environmental conditions. Moreover, this finding might open a new avenue for oleaginous yeast control and modification for further industrial applications.

## Methods

### Yeast strain, cultivation, and sample collection

*Yarrowia lipolytica* PO1d obtained from the National University of Singapore were pre-cultivated for 17 h in synthetic complete (SC) medium (2%w/v glucose, 0.64%w/v yeast nitrogen-based without amino acid, 0.19%w/v drop-out mix without uracil, 0.00076%w/v uracil) in 100 mL Erlenmeyer flasks at the constant temperature of 30 °C and the shaking speed of 200 rpm. The cells were then inoculated into 20 mL SC medium without ammonium sulfate (nitrogen limiting condition) to obtain the initial optical density at 600 nm of 0.1 in 100 mL Erlenmeyer flasks at the constant temperature of 30 °C and the shaking speed of 200 rpm. For the sample collection, 5 OD_600_ units of cells were collected at 36 h by means of fast filtration using the 0.45 µm pore size, 47 mm diameter nylon membrane (Millipore). The cells were then immersed in liquid nitrogen immediately for metabolism quenching and kept at -80 °C until extraction. For extraction, 1.8 mL of extraction solvent (methanol/water/chloroform = 5:2:2 v/v/v, with 20 µg/L of ( +)-10 camphorsulfonic acid as an internal standard) was added to each 2-mL sampling tube with filtered sample and incubated at -30 °C for 1 h. After incubation, 700 µL of the solution was transferred to a new tube containing 350 µL of water. The mixture was mixed using a vortex and centrifuged at 16,000 × g for 3 min at 4 °C to separate polar and non-polar phases. 700 µL of the upper polar phase was transferred to a new tube via syringe filtration (0.2 µm PTFE hydrophilic membrane, Millipore). The sample was centrifugally concentrated for 2 h and freeze-dried overnight. After reconstituting in 100 µL ultra-pure water, the sample was centrifuged at 16,000 × g for 3 min at 4 °C and transferred to an LC glass vial. All experiment was done in triplicates.

### LCMS analysis for MEP

The MEP authentic standard was purchased from Sigma-Aldrich (product number 52131). The samples were analyzed based on the method adopted from Dempo et al.^43^ using a Nexera UHPLC system (Shimadzu, Kyoto, Japan) coupled with LC/QqQ/MS 8030 Plus (Shimadzu). The column used was CERI ODS L-column 2 metal free column (150 mm × 2.1 mm, particle size 3 µm), the mobile phase was (A): 10 mM tributylamine and 15 mM acetate in ultra-pure water, (B): methanol. The flow rate was set to 0.2 mL/min, the column oven temperature was set to 45 °C. The mobile phase (B) was increased from 0 to 15%, 50%, and 100% from 1.0 to 1.5 min, 3.0 to 8.0 min, and 8.0 to 10.0 min, respectively; held until 11.5 min, decreased to 0% from 11.5 min and held at 0% until 20 min. The analysis mode was negative ion detection mode. The injection volume was 3 µL, probe position was + 1.5 mm, desolvation line temperature was 250 °C, heat block temperature was 400 °C, nebulizer gas flow was 2 L/min, and drying gas flow was 15 L/min. All analysis was done in triplicates.

### Effect of MEP pathway inhibitor analysis

To assess the DXR activity, the enzyme specific to the MEP pathway in *Y. lipolytica*, DXP reductoisomerase inhibitor (Fosmidomycin) was used. Fosmidomycin sodium salt hydrate ≥ 95% (NMR) was purchased from Sigma-Aldrich (product number F8682). *Yarrowia lipolytica* the main culture, was cultivated and collected as mentioned above, using medium with limited nitrogen source with the addition of 100 µM inhibitor. The samples were analyzed using the LCMS method above. All analysis was done in triplicates.

### 1-^13^C carbon isotope glucose labeled analysis

To further assess if the MEP pathway was working in *Y. lipolytica*, we analyze ergosterol in *Y. lipolytica,* which is the natural end product derived from DMAPP. The 1-^13^C D-glucose (98–99%) was purchased from Cambridge Isotope Laboratories (Item Number CLM-420-PK). *Yarrowia lipolytica* PO1d were pre-cultivated overnight in synthetic complete (SC) medium (2%w/v glucose, 0.64%w/v yeast nitrogen-based without amino acid, 0.19%w/v drop-out mix without uracil, 0.00076%w/v uracil) in 100 mL shake flasks at 30 °C and 200 rpm. The pre-cultured cells were inoculated into 20 mL 10% 1-^13^C D-glucose SC medium without ammonium sulfate to obtain the initial optical density at 600 nm of 0.1 in 100 mL shake flasks at 30 °C and 200 rpm. For sample collection, the cell pellet was collected at 36 h by centrifugation at 4 °C, 10,000 rpm, 10 min. The cell pellet was then submerged in liquid nitrogen and subjected to lyophilization overnight for further use. For extraction, 1 g of lyophilized cell pellet was extracted by 20 mL of extraction solvent (chloroform: methanol = 2:1 v/v, with 20 µg/L) for 2 h with stirring. After incubation, the solution was filtered with filter paper with cut-off diameter of 3 µM (Advantech No.6), the extraction was repeated three times. The solution was evaporated until dryness using a rotary evaporator. The ergosterol was then separated from the crude extract by open column chromatography; the fraction containing ergosterol was confirmed by GCMS (Supplementary Fig. 7). 0.1 mg. of dried ergosterol fraction was dissolved in 1 mL methanol then analyze using a Nexera UHPLC system (Shimadzu, Kyoto, Japan) coupled with LC/QqQ/MS 8050 (Shimadzu) in positive ion detection mode. The column used was inertSustain AQ-C18 3 µm 2.1 × 150 mm (GL science); the mobile phase was methanol: acetonitrile 80:20, flow rate: 0.2 mL/min, oven temperature: 40 °C, injection volume 3 µL, LC program: isocratic flow. The collision energy optimized for ergosterol fragmentation at q2 was -25 eV. All analysis was done in triplicates, for qualitative analysis , the representative sample was selected for discussion.

## Supplementary Information

Supplementary Information 1.

Supplementary Information 2.

Supplementary Information 3.

Supplementary Information 4.

Supplementary Information 5.

Supplementary Information 6.

Supplementary Information 7.

Supplementary Information 8.

Supplementary Information 9.

Supplementary Information 10.

Supplementary Information 11.

Supplementary Information 12.

Supplementary Information 13.

Supplementary Information 14.

Supplementary Information 15.

Supplementary Information 16.

Supplementary Information 17.

Supplementary Information 18.

Supplementary Information 19.

Supplementary Information 20.

Supplementary Information 21.

Supplementary Information 22.
