# Supplementary material for: Stable isotope and chemical inhibition analyses suggested the existence of a non-mevalonate-like pathway in the yeast Yarrowia lipolytica
Source: Sci Rep. 2021 Mar 10;11:5598. doi: 10.1038/s41598-021-85170-0 (PMC7970925; doi:10.1038/s41598-021-85170-0)
Supplement: Supplementary file 1 — Supplementary Information 1. [file 41598_2021_85170_MOESM1_ESM.docx]

**Stable isotope and chemical inhibition analyses suggested the existence of a non-mevalonate-like pathway in the yeast *Yarrowia lipolytica* Supplementary data**

Sivamoke Dissook^1^

Email: sivamoke@bio.eng.osaka-u.ac.jp

Tomohisa Kuzuyama^2^

Email: utkuz@g.ecc.u-tokyo.ac.jp

Yuri Nishimoto^3^

Email: yuri.nishimoto0727@gmail.com

Shigeru Kitani^3^

Email: kitani@icb.osaka-u.ac.jp

Sastia Putri^1*^

Email: sastia_putri@bio.eng.osaka-u.ac.jp

Eiichiro Fukusaki^1^

Email: fukusaki@bio.eng.osaka-u.ac.jp

*Corresponding author

Phone/Fax: +81-6-6879-7424

^1^Department of Biotechnology, Graduate School of Engineering, Osaka University, 2-1 Yamadaoka, Suita, Osaka 565-0871, Japan.

^2^Graduate School of Agricultural and Life Sciences, The University of Tokyo, 1-1-1 Yayoi, Bunkyo-ku, Tokyo 113-8657, Japan.

^3^International Center for Biotechnology, Osaka University, 2-1 Yamadaoka, Suita, Osaka 565-0871, Japan.


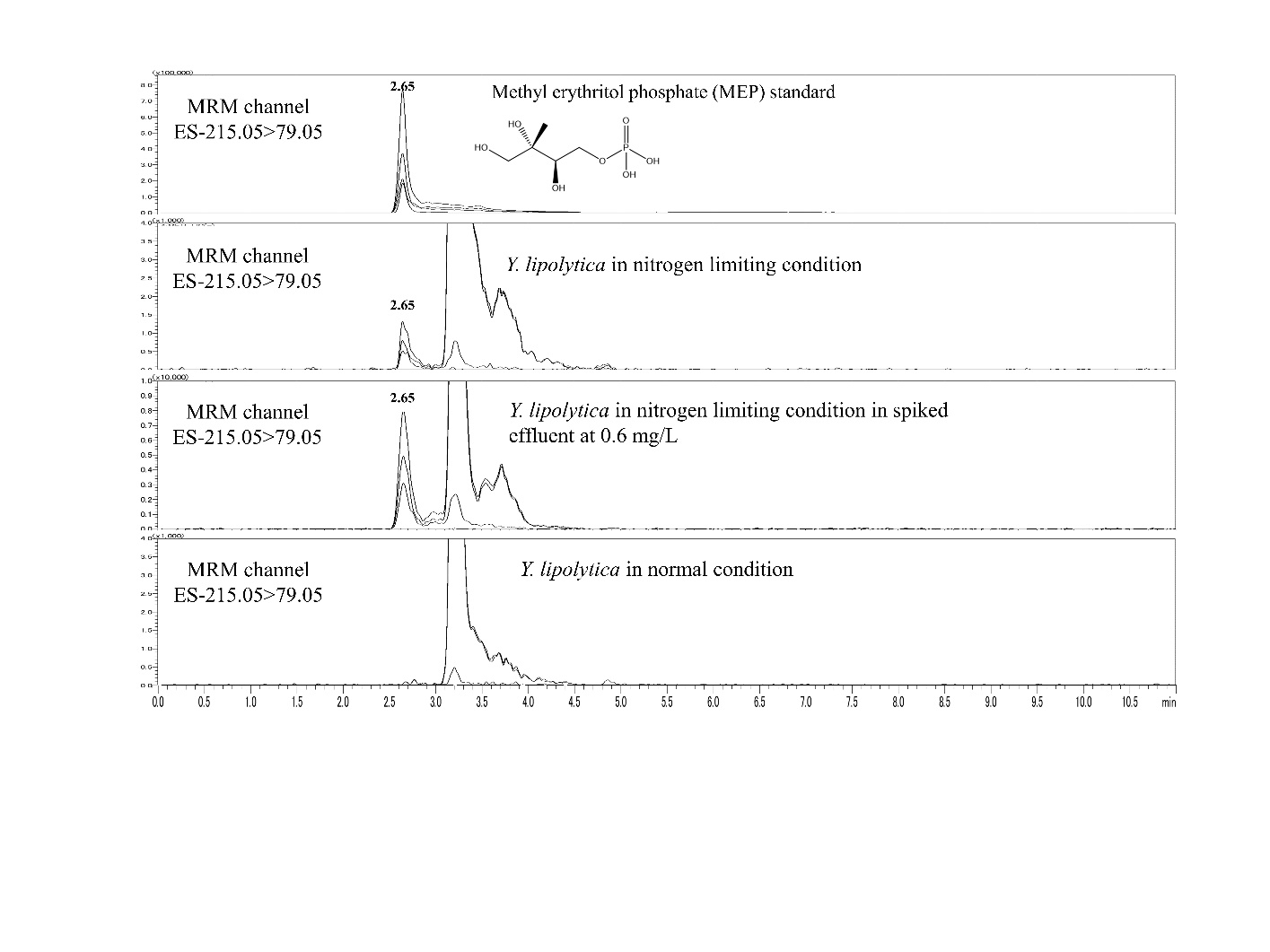


**Supplementary Fig. 1**: MEP analysis results from the different analytical systems.

We took extra steps to verify the MEP annotation by performing the analysis in different cultivation batch, machines, and columns. In this case, the system’s configuration was a Nexera UHPLC system (Shimadzu, Kyoto, Japan) coupled with LC/QqQ/MS 8050 (Shimadzu) in negative ion detection mode. The column used was RESTEK FluoroPhenyl metal-free (100 mm × 2.1 mm, particle size 2.7 μm); the mobile phase was (A): 10 mM ammonium acetate in ultra-pure water (B): 100 % acetonitrile. The figure shows multiple reaction monitoring (MRM) chromatograms of methyl erythritol phosphate (MEP) standard, *Y. lipolytica* in nitrogen limiting condition, *Y. lipolytica* in nitrogen limiting condition in spiked effluent at 0.6 mg/L, and *Y. lipolytica* in normal conditions.

**
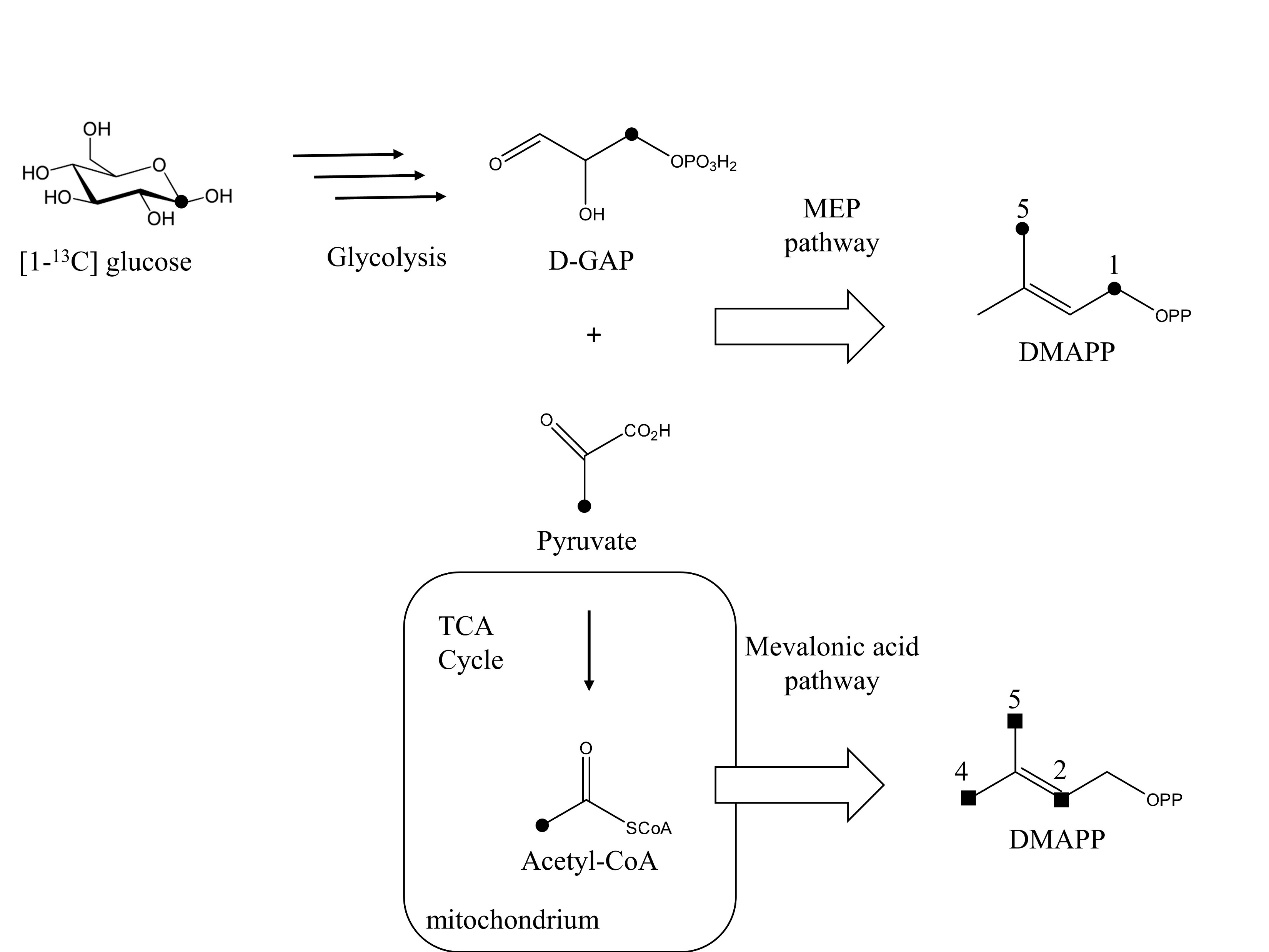
Supplementary Fig. 2.**

Incorporation of [1-13C] glucose into isoprenoids via the MEP pathway and the MVA pathway. In the MEP pathway, DMAPP is biosynthesized from one molecule of D-GAP and one molecule of pyruvate, each of which is derived from one molecule of glucose; one molecule of DMAPP is derived from two molecules of glucose. Therefore, the ^13^C atoms derived from [1-13C] glucose are incorporated at C1 and C5 of DMAPP. In the MVA pathway, DMAPP is biosynthesized from three molecules of acetyl-CoA, which are derived from three molecules of glucose; one molecule of DMAPP is derived from three molecules of glucose. Therefore, the ^13^C atoms derived from [1-13C] glucose are incorporated at C2, C4, and C5 of DMAPP.


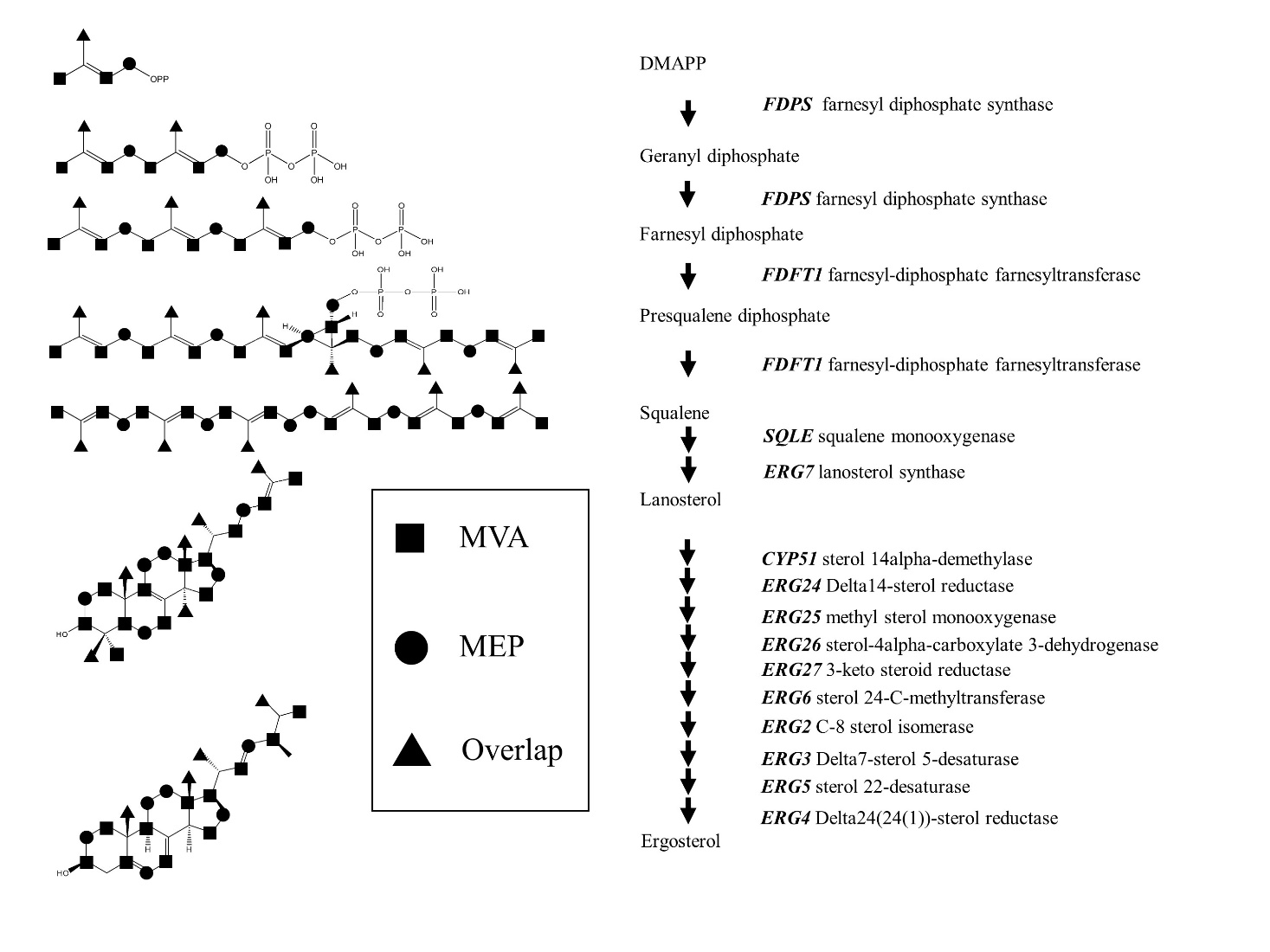


**Supplementary Fig. 3.**

13C incorporation into ergosterol; the circles indicate the theoretically labeled position by the MEP pathway, the squares indicate the theoretically labeled position by the MVA pathway, the triangles indicate the theoretically labeled position by both pathways.

**Supplementary Table 1.**

The product ion *m/z* from fragment analysis of ergosterol.

| Nitrogen limiting C12 | | Nitrogen limiting C13 | | Normal C12 | | Normal C13 | |
| --- | --- | --- | --- | --- | --- | --- | --- |
| m/z | intensity | m/z | intensity | m/z | intensity | m/z | intensity |
| 41 | 256554 | 39 | 1855 | 41 | 110237 | 55 | 2491 |
| 55 | 353476 | 41 | 2378 | 55 | 112282 | 57 | 5178 |
| 57 | 1136353 | 57 | 6959 | 57 | 260743 | 59 | 1740 |
| 69 | 3868992 | 60 | 3711 | 69 | 1177071 | 69 | 4425 |
| 81 | 1171627 | 67 | 2378 | 81 | 256293 | 70 | 7226 |
| 83 | 1379537 | 69 | 8117 | 83 | 332143 | 71 | 1855 |
| 93 | 290600 | 70 | 6444 | 93 | 137154 | 77 | 1855 |
| 95 | 488329 | 71 | 5757 | 95 | 224395 | 81 | 4302 |
| 97 | 198103 | 82 | 4640 | 97 | 62890 | 82 | 4222 |
| 105 | 586472 | 84 | 1582 | 105 | 138456 | 93 | 927 |
| 107 | 547729 | 95 | 1855 | 107 | 263943 | 95 | 3711 |
| 109 | 451180 | 96 | 1855 | 109 | 162104 | 98 | 5568 |
| 117 | 255809 | 96 | 5568 | 117 | 87145 | 105 | 1855 |
| 119 | 459903 | 107 | 1771 | 119 | 121133 | 107 | 3401 |
| 121 | 587729 | 109 | 3711 | 121 | 112940 | 108 | 1855 |
| 123 | 420763 | 120 | 1855 | 123 | 127666 | 109 | 11489 |
| 125 | 206473 | 121 | 3711 | 125 | 64215 | 111 | 927 |
| 131 | 569455 | 124 | 1855 | 129 | 105519 | 117 | 4306 |
| 133 | 696790 | 134 | 2658 | 131 | 185720 | 118 | 1855 |
| 135 | 369252 | 136 | 3711 | 133 | 201354 | 122 | 1855 |
| 143 | 452733 | 143 | 12547 | 135 | 176848 | 124 | 2699 |
| 145 | 1553569 | 145 | 2783 | 143 | 176650 | 125 | 1771 |
| 147 | 775393 | 146 | 3711 | 145 | 582841 | 127 | 3711 |
| 149 | 582122 | 147 | 7426 | 147 | 238894 | 131 | 1855 |
| 157 | 979845 | 148 | 8616 | 149 | 106869 | 132 | 1855 |
| 159 | 1943788 | 152 | 3932 | 157 | 211179 | 133 | 2783 |
| 160 | 319400 | 157 | 2783 | 159 | 530682 | 145 | 1855 |
| 161 | 591147 | 159 | 7278 | 161 | 181820 | 149 | 2491 |
| 163 | 231201 | 160 | 9501 | 163 | 71896 | 150 | 1855 |
| 171 | 567326 | 161 | 7363 | 171 | 174409 | 159 | 1855 |
| 173 | 446233 | 162 | 1855 | 173 | 144025 | 164 | 1855 |
| 183 | 297834 | 163 | 3377 | 175 | 77528 | 166 | 3597 |
| 185 | 490710 | 172 | 1771 | 177 | 65388 | 172 | 1855 |
| 187 | 361713 | 173 | 7426 | 183 | 85516 | 185 | 1855 |
| 197 | 300844 | 177 | 2158 | 185 | 222970 | 186 | 2878 |
| 199 | 445516 | 184 | 3711 | 187 | 214157 | 188 | 2783 |
| 201 | 463306 | 187 | 4431 | 191 | 69213 | 193 | 3711 |
| 211 | 327388 | 188 | 1839 | 197 | 144018 | 198 | 3711 |
| 213 | 628305 | 200 | 4139 | 199 | 322460 | 199 | 2783 |
| 225 | 418192 | 201 | 1855 | 201 | 188933 | 200 | 3711 |
| 227 | 260777 | 206 | 3711 | 203 | 65473 | 201 | 2158 |
| 239 | 250317 | 216 | 2491 | 211 | 83486 | 202 | 3711 |
| 253 | 657300 | 217 | 1855 | 213 | 289662 | 211 | 1855 |
| 255 | 242392 | 225 | 2783 | 225 | 114123 | 212 | 3711 |
| 281 | 238069 | 240 | 1855 | 227 | 59420 | 216 | 3628 |
| 295 | 724590 | 244 | 1855 | 229 | 65283 | 225 | 3711 |
| 309 | 481630 | 245 | 1855 | 239 | 197344 | 227 | 3711 |
| 379 | 1873541 | 256 | 3723 | 241 | 61220 | 228 | 8253 |
| 380 | 642199 | 257 | 1855 | 253 | 287472 | 241 | 927 |
|  |  | 258 | 7258 | 267 | 88930 | 242 | 7426 |
|  |  | 275 | 1855 | 269 | 58872 | 248 | 927 |
|  |  | 284 | 3711 | 281 | 134583 | 255 | 1855 |
|  |  | 296 | 5568 | 295 | 269126 | 257 | 6204 |
|  |  | 301 | 1855 | 297 | 67253 | 261 | 3711 |
|  |  | 312 | 8525 | 309 | 160762 | 283 | 1855 |
|  |  | 313 | 3711 | 323 | 98457 | 295 | 927 |
|  |  | 337 | 2878 | 379 | 940153 | 307 | 1855 |
|  |  | 383 | 20654 |  |  | 311 | 1855 |
|  |  |  |  |  |  | 312 | 3711 |
|  |  |  |  |  |  | 325 | 5568 |
|  |  |  |  |  |  | 366 | 1188 |
|  |  |  |  |  |  | 383 | 2726 |

The list of product ion derived from ergosterol fragmentation. “Nitrogen limiting C12”, ergosterol from the yeast cells cultivated in nitrogen limiting condition; “Nitrogen limiting C13”, ergosterol from the yeast cells cultivated in the presence of [1-^13^C] glucose in nitrogen limiting condition; “Normal C12”, ergosterol from the yeast cells cultivated in normal condition; “Normal C13”, ergosterol from the yeast cells cultivated in the presence of [1-^13^C] glucose in normal condition.


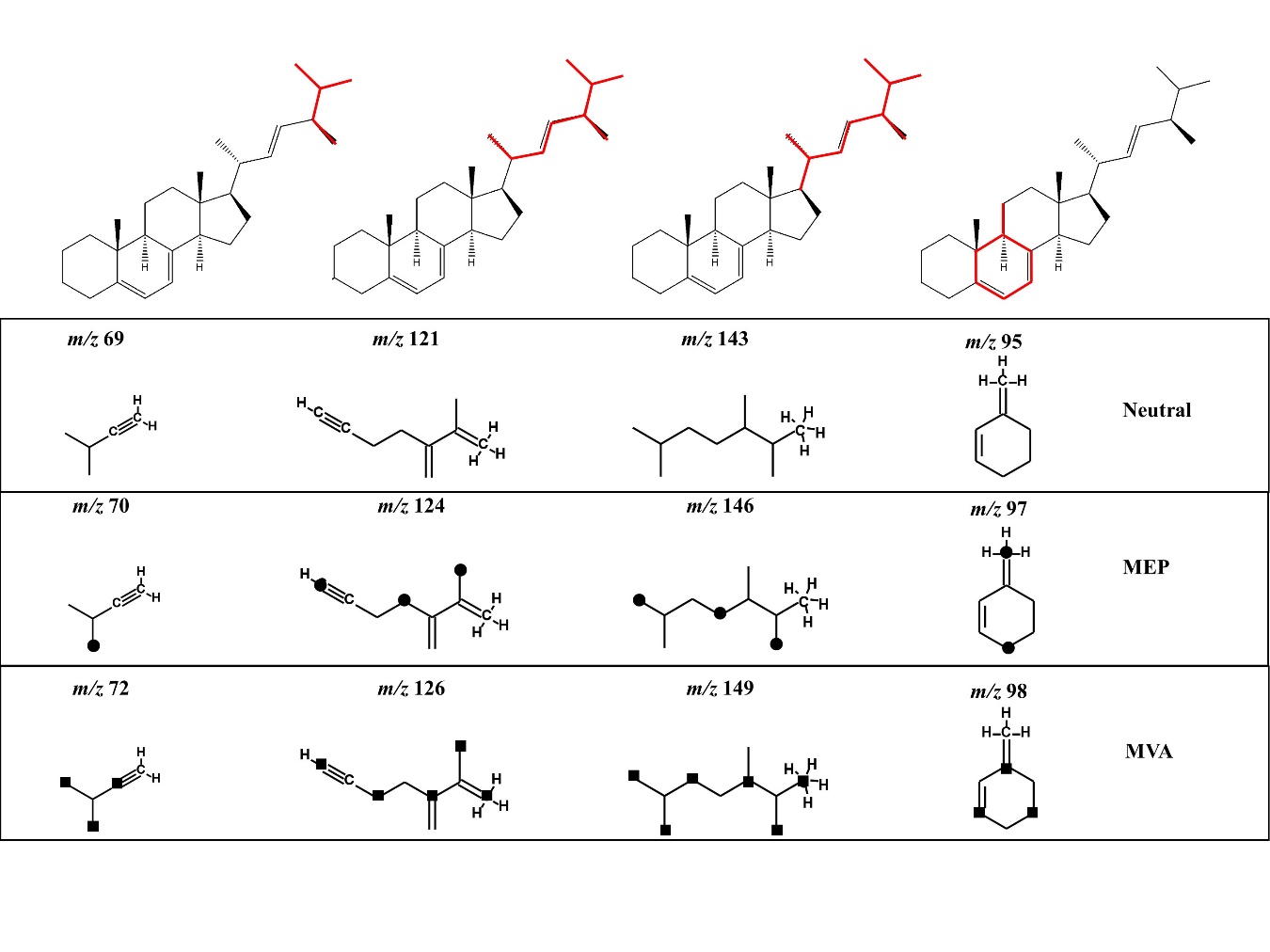


**Supplementary Fig. 4.**

The predicted fragment *m/z* and labeled positions.

**Supplementary 1**: MEP pathway gene candidate search based NCBI BLAST.

To assess the genetics potential for the MEP pathway in *Y. lipolytica*, an in-house bioinformatics workflow was developed. In this study, a custom database for each gene in the MEP pathway was built. The consensus conserved protein domain sequence of genes in the MEP pathway was retrieved from NCBI. The retrieved sequence was aligned against the NCBI non-redundant protein database using NCBI blast+ v.2.7.1 (standalone). The protein sequences that produce a significant alignment with e-value less than 10e-6 were extracted to construct the MEP pathway enzymes database. The reference genome of *Y. lipolytica* was retrieved from the NCBI FTP site in GenBank format. The genome file was processed using an in-house processing program written in Perl programming language results in 6,448 protein sequences, 6,950 DNA sequences, and 6 chromosome sequences. All of the protein sequences from *Y. lipolytica* genome was compared against the MEP pathway enzyme database constructed earlier using NCBI blast+ v.2.7.1 (standalone). The protein sequences that produce a significant alignment with e-value less than 10e-6 and have percent identity greater or equal to 25 percent^37^ were extracted for further alignment by global alignment algorithm using CLUSTALW 2.0 software^38^. Top non-redundant *Y. lipolytica* genes that align with genes from plants or prokaryotes were select as candidate genes for the MEP pathway in *Y. lipolytica.* Although we found some candidate alignment but none of our candidates contain NADPH binding site domain (GSTGSIG), which is essential for Dxr activity, therefore the gene responsible for the Dxr-like activity in *Y. lipolytica* is still unclear. The candidate gene sequence and the subject sequence in fasta format could be found in the online supplementary data.


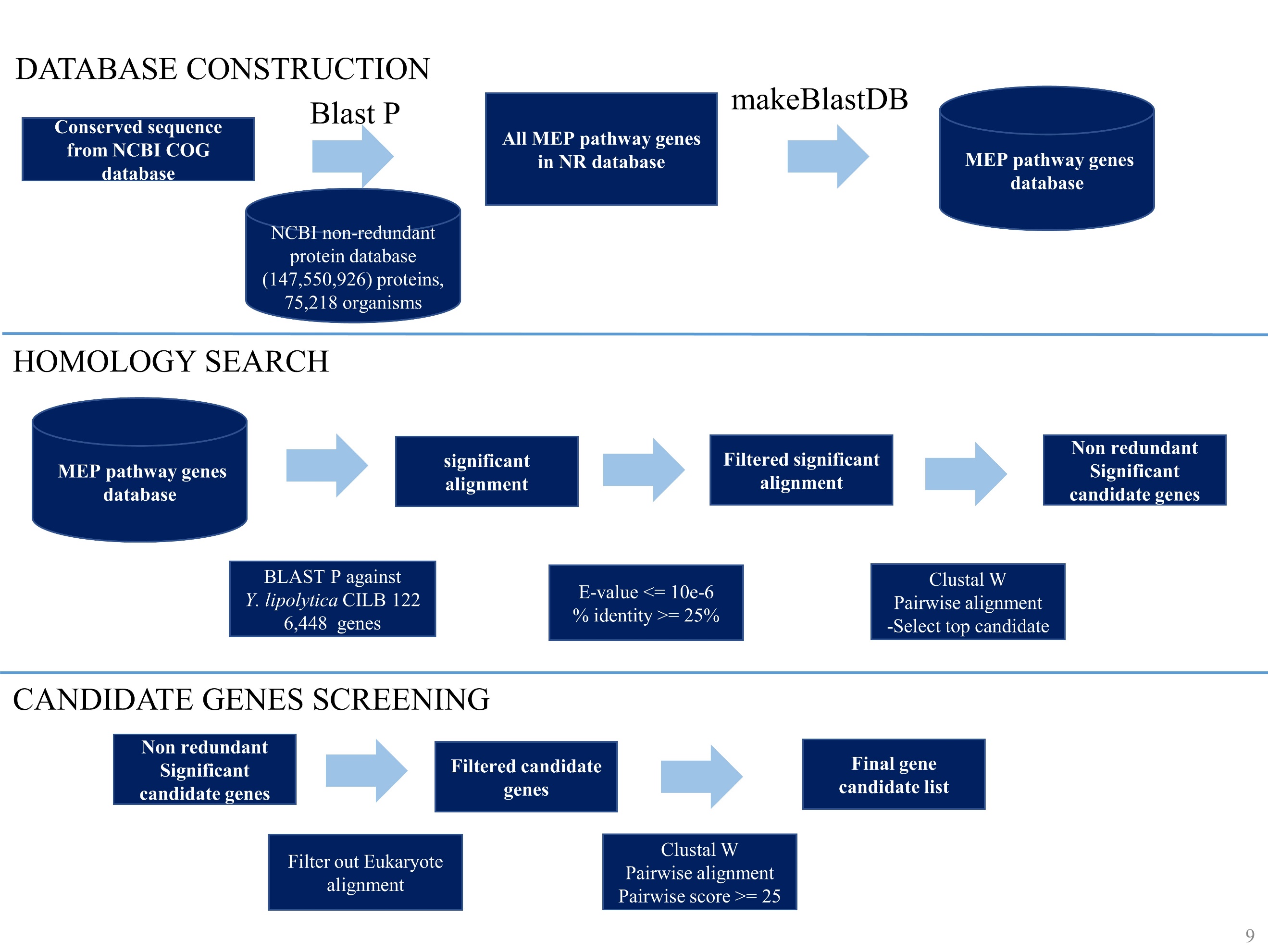
**Supplementary Fig. 5.**

Illustration of the bioinformatics workflow described in Supplementary 1.

**Supplementary 2**: MEP pathway gene candidate based on previously published RNA sequencing data analysis. To search for the potential MEP pathway gene, we review the published RNA sequencing data in which the *Y. lipolytica* was subjected to the nitrogen limiting condition. The closest data set we found was that of Kerkhoven, Eduard J., *et al.*(2017) [45]. This study compares the nitrogen limiting condition with the carbon limiting condition, ideally the comparison between the nitrogen limiting condition and standard condition; however, for the purpose of extracting the differentially expressed genes during the nitrogen limiting condition, any other different condition would be useful. We start by mapping the raw reads from the array express archive number E-MTAB-5284 to the reference *Y. lipolytica* genome assembly accession: GCF_000002525.2 using HISAT2 [46]. Then we use StringTie [47] to assemble the alignment into the potential transcripts. Finally, we calculate the significantly different gene expression using the DESeq2 package [48]. To narrow down the significantly expressed genes, we compare the protein motif from the significantly expressed genes in nitrogen limiting condition to that of the genes from the MEP pathway. We utilized the MEME (Multiple EM for Motif Elicitation) [49] software to extract the motif information from six gene clusters in the MEP pathway listed in the NCBI COG database, namely DXS, DXR, ispD, ispE, ispF, ipsG, and ipsH. Then we scan the significantly expressed genes in nitrogen limiting condition with the motif information obtained from the earlier step using FIMO software [50]. The candidate gene sequence and the subject sequence in fasta format could be found in the online supplementary data.


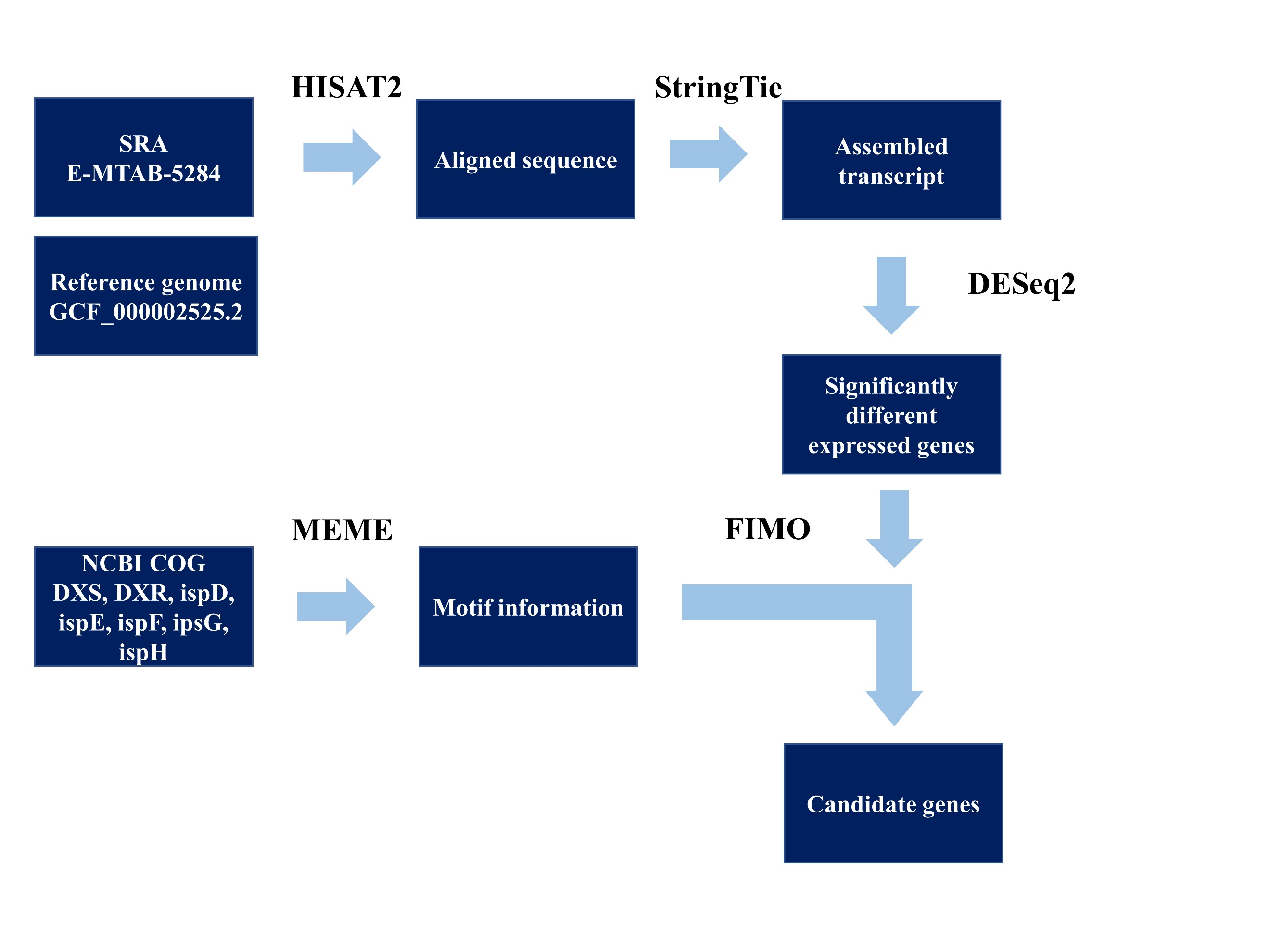


**Supplementary Fig. 6.**

Illustration of the bioinformatics workflow described in Supplementary 2.


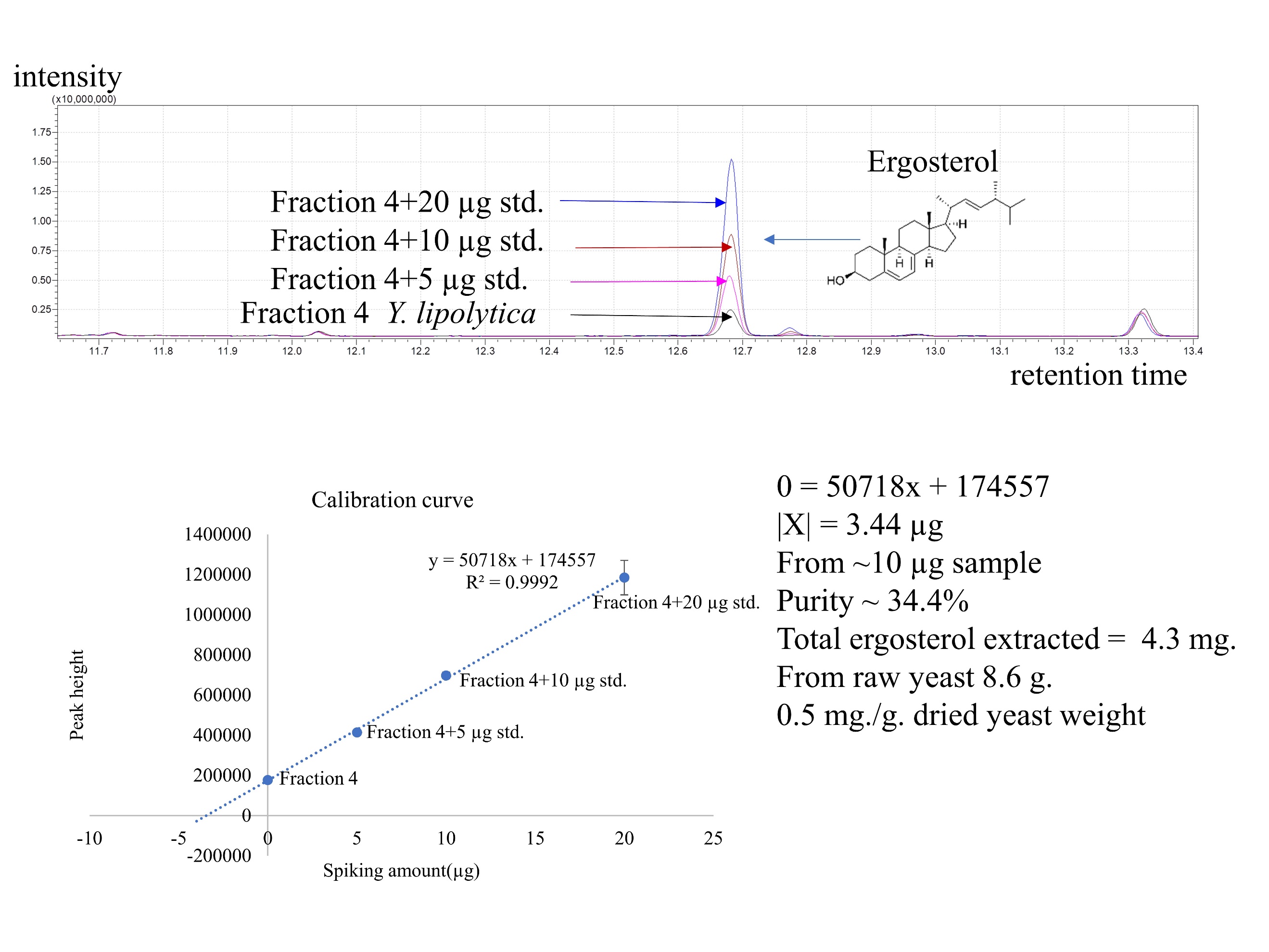


**Supplementary Fig. 7**: Extraction and identification of ergosterol from *Y. lipolytica* using GC/MS and the authentic standard spiking. The calibration curve is also shown to determine the amount of ergosterol extracted from the cells of *Y. lipolytica*. Fraction 4 indicates the name of the fraction from the open column chromatography that contains ergosterol.
